# Tumour‐associated macrophages in gastric cancer: From function and mechanism to application

**DOI:** 10.1002/ctm2.1386

**Published:** 2023-08-22

**Authors:** Jie Li, Juan Sun, Ziyang Zeng, Zhen Liu, Mingwei Ma, Zicheng Zheng, Yixuan He, Weiming Kang

**Affiliations:** ^1^ Department of General Surgery Peking Union Medical College Hospital Chinese Academy of Medical Sciences & Peking Union Medical College Beijing People's Republic of China

**Keywords:** gastric cancer, immune infiltration, macrophage polarisation, prognostic prediction, tumour‐associated macrophage

## Abstract

**Background:**

Gastric cancer (GC) is a malignant tumour, with high morbidity and mortality rates worldwide. The occurrence and development of GC is a complex process involving genetic changes in tumour cells and the influence of the surrounding tumour microenvironment (TME). Accumulative evidence shows that tumour‐associated macrophages (TAMs) play a vital role in GC, acting as plentiful and active infiltrating inflammatory cells in the TME.

**Main body:**

In this review, the different functions and mechanisms of TAMs in GC progression, including the conversion of phenotypic subtypes; promotion of tumour proliferation, invasion and migration; induction of chemoresistance; promotion of angiogenesis; modulation of immunosuppression; reprogramming of metabolism; and interaction with the microbial community are summarised. Although the role of TAMs in GC remains controversial in clinical settings, clarifying their significance in the treatment selection and prognostic prediction of GC could support optimising TAM‐centred clinicaltherapy.

**Conclusion:**

In summary, we reviewed the the phenotypic polarisation, function and molecular mechanism of TAMs and their potential applications in the treatment selection and prognostic prediction of GC.

## Graphical abstract

- Tumour‐associated macrophages (TAMs) promote gastric cancer (GC) progression by stimulating angiogenesis; promoting cell proliferation, invasion and metastasis; inhibiting the anti‐tumour immune response; conferring chemoresistance; regulating metabolism; and interacting with the microbiota in the stomach.
- TAMs can be used as a potential target in treatment of GC.
- The infiltrations of TAMs predict the prognoses of GC patients.

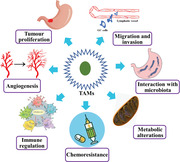

## 1 INTRODUCTION

Gastric cancer (GC) is one of the most frequent malignancies worldwide, with global morbidity and cancer‐related mortality ranked fifth and fourth, respectively.^1^ Moreover, new GC cases in China account for 44.1% of the global new cases annually. Meanwhile, GC‐associated deaths in China also account for more than 50% of the total global deaths from GC. Thus, GC significantly affects patients’ health along with subjecting them to significant economic and mental burdens.^2^

The risk factors for GC mainly contain *Helicobacter pylori* infections, heredity, unhealthy diets and environmental pollution. Intracellular genetic and epigenetic alterations, such as the mutation of oncogenes and the activation of abnormal proliferative signals from the tumour microenvironment (TME), act an intrinsic role in GC progression. *H. pylori* infections and unhealthy lifestyles, such as lack of exercise, smoking and drinking and excessive intake of salt, constitute the exogenous risk factors for GC, which further cooperate with endogenous factors to aggravate GC progression.^3^, ^4^

Accumulating research shows that the TME, involving the genetic and epigenetic changes of tumour cells, also acts key roles in the tumourigenesis, progression and metastasis of tumours. The components of the TME include all non‐malignant stromal cells surrounding the tumour cells, such as fibroblasts, smooth muscle cells, blood cells and immune cells.^5^, ^6^ In 1889, the ‘seed and soil’ theory of tumour metastasis was proposed. On investigating the ‘soil’ mainly composed of tumour cells and immune‐related cells, it was observed that related immunity cells express a specific biological phenotype in the TME through interaction with cancer cells.^7^, ^8^, ^9^, ^10^ Therefore, the TME was hypothesised to be a unique and dynamic environment that develops and changes with tumour progression, significantly affecting tumour development. It is speculated that ignoring the dynamic changes of the TME during tumour progression is one of the important reasons for the poor results of comprehensive therapy against tumour cells. Hence, the conventional treatment is combined with precise treatment strategy, focusing on the dynamic cellular changes in the TME, to benefit patients with GC in comprehensive medical treatment.

Tumour‐associated macrophages (TAMs), the key effector cells of innate immunity, act a key role in the immune microenvironment of tumours.^11^, ^12^ TAMs can be divided into M1 and M2 types. M1 type TAMs mainly exert anti‐tumour effects, while M2 type TAMs promote tumour progression by interleukin (IL)‐10 and transforming growth factor‐β (TGF‐β).^13^ Additionally, studies report that the mutual promotion among tumour cells and TAMs significantly affects tumour progression.^14^ However, the role of TAMs in GC is more complex, and reprogramming the polarisation of TAMs could contribute to the advancement of tumour immunotherapy.^15^, ^16^ This review summarises the phenotypic polarisation of TAMs, the interactions between TAMs and diverse cell types in TME and the potential functions and mechanisms of TAMs in GC. Furthermore, it also reviews how these functions and mechanisms can be utilised as prognostic biomarkers for patients with GC and potential targets for GC treatment selection.

## 2 TAMS ORIGIN AND PHENOTYPIC POLARISATION

Macrophages are specialised, long‐living, phagocytic cells of the innate immune system and the first responders to infections.^17^ Macrophages exist in most tissues, wherein they not only initiate an immune response and inflammatory response to pathogens but also act a key role in regulating tissue homeostasis and tissue repair and remodelling.^18^ Macrophages residing in tissues are mainly differentiated from circulating monocytes,^19^ which in turn are mainly derived from haematopoietic stem cells in the bone marrow and can also be generated in embryonic areas near the foetal liver, yolk sac or dorsal aorta during embryonic development.^20^ Circulating monocytes migrate to various tissues in a stable state or during inflammation and then differentiate into persistent tissue‐specific macrophages, including osteoclasts, microglia cells, histiocytes, Kupffer cells and alveolar macrophages.^21^ Furthermore, changes in different signalling molecules can lead to different phenotype secretion and macrophage activation states.^22^, ^23^

Macrophages can be divided into two subtypes according to their functions and effects after activation: classically activated M1 macrophages and alternatively activated M2 macrophages. M1 macrophages exert functions in type 1 helper T (Th1) cell recruitment, pathogen resistance and tumour killing mainly through natural and adaptive immune responses.^24^ Additionally, M1 macrophages are usually induced by pathogens, lipopolysaccharide, GM‐CSF, tumour necrosis factor‐α (TNF‐α), Th1 cytokines and interferon‐γ (IFN‐γ).^25^ M2 macrophages are usually activated by parasites or fungi, immune complexes, apoptotic cells, macrophage‐colony stimulating factor (M‐CSF), IL‐13, TGF‐β and Th2 cells.^26^, ^27^ The four subtypes of M2 macrophages are based on their different stimuli: M2a (activated by IL‐4 and IL‐13), M2b (activated by immune complexes, Toll‐like receptor ligands and IL‐1β), M2c (activated by glucocorticoids, IL‐10 and TGF‐β) and M2d (activated by IL‐6 and adenosine).^28^, ^29^ Notably, M1 and M2 polarisation states are not immutable and can be converted into each other, thus they can be used as a potential target for GC immunotherapy.^30^, ^31^

## 3 THE INTERACTIONS BETWEEN TAMS AND DIVERSE CELL TYPES IN TME

**FIGURE 1:**
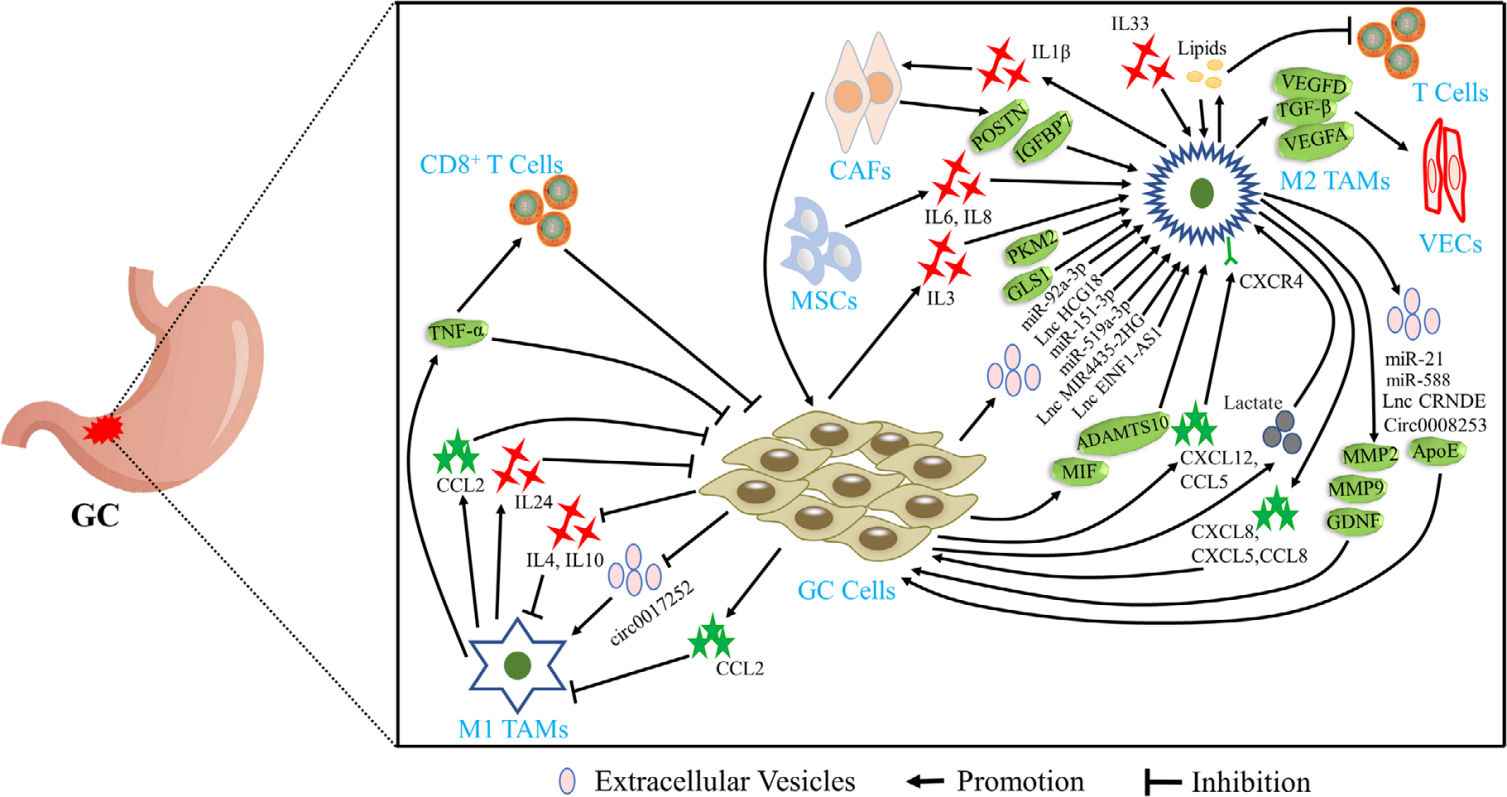
Tumour‐associated macrophages (TAMs) interplay with diverse types of cells in tumour microenvironment (TME) of gastric cancer (GC). CAFs, cancer‐associated fibroblasts; IL, interleukin; MSCs, mesenchymal stem cells; VECs, vascular endothelial cells.

The components of TME are diverse and complex. All non‐malignant mesenchymal cells around tumour cells, such as fibroblasts, smooth muscle cells, endothelial cells and various immune cells, interact with each other and jointly affect tumour progression. TAMs interplay with diverse types of cells in TME through the secretion of extracellular vesicles (EVs), cytokines, chemokines and proteins, including tumour cells, T cells, vascular endothelial cells (VECs), cancer‐associated fibroblasts (CAFs) and mesenchymal stem cells (MSCs), thereby facilitating GC progression. As shown in Figure 1, cumulative studies have demonstrated that tumour cells can affect the dominant type of TAMs by secreting different communicative mediators, and then construct a microenvironment that is most suitable for tumour cell progression. Current research has revealed that M1 TAMs inhibited the cell viabilities and enhanced cell chemosensitivity of GC by secreting IL‐24, C‐C motif chemokine ligand (CCL)2 and TNF‐α.^16^, ^32^, ^33^ M2 TAMs, which occupy the dominant position in TME, exert their roles on tumour cells by secreting EVs (miR‐21,^34^ miR‐588,^35^ lncRNA CRNDE,^36^ circ 0008235^37^ and ApoE^38^), proteins (MMP2^39^ and MMP9^40^), various chemokines (CXCL5,^41^ CXCL8^42^ and CCL8^43^) and reprogramming metabolism, thereby promoting cell proliferation and metastasis, conferring chemoresistance, and ultimately aggravating the progression of GC.^44^, ^45^, ^46^, ^47^ In addition, M1 TAMs enhanced the immune clearance of tumour cells via boosting the performance of CD8^+^ T cells by increasing the secretions of TNF‐α in GC.^33^ However, M2 TAMs inhibited the functions of T cells by increasing the accumulation of lipids in TME, thereby causing immune evasion of GC cells.^44^ On the one hand, CAFs triggered phosphatidylinositol‐3‐kinase/protein kinase B (PI3K/Akt) pathway to induce the polarisation of M2 TAMs by secreting IGFBP7 and POSTN.^48^, ^49^ On the other hand, mixed TAMs of M1 and M2 enhanced the cancer‐promoting roles of CAFs by increasing the secretion of IL‐1β in TME, which accelerated the malignant progression of GC.^50^ Moreover, MSCs derived from GC activated the JAK2/STAT3 pathway to enhance the polarisation of M2 TAMs by increasing the secretion of IL‐6 and IL‐8.^51^ Furthermore, M2 TAMs promoted the viabilities of VECs by increasing the contents of VEGFA, VEGFD and TGF‐β in TME, resulting in the rapid growth and distant metastasis of GC.^52^ Abovementioned research revealed that TAMs interplay with diverse types of cells in TME, resulting in jointly affecting GC progression.

## 4 THE ROLE AND MECHANISM OF TAMS IN GC

**FIGURE 2:**
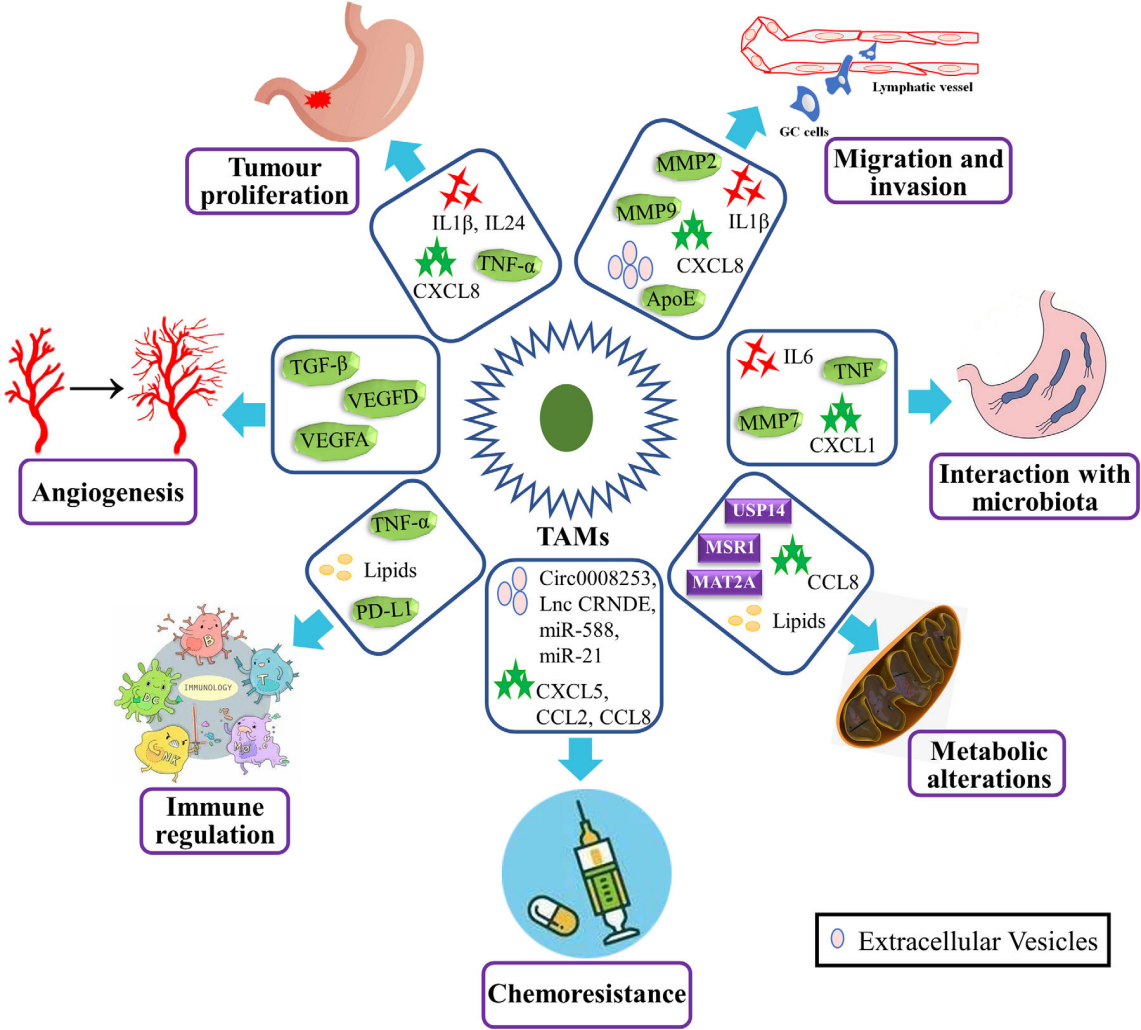
Tumour‐associated macrophages (TAMs) promote gastric cancer (GC) progression by stimulating tumour‐related angiogenesis; promoting cell proliferation, invasion and metastasis; inhibiting the anti‐tumour immune response; conferring chemoresistance; regulating metabolism; and interacting with the microbiota in the stomach. IL, interleukin; TGF‐β, transforming growth factor‐β; TNF‐α, tumour necrosis factor‐α.

Macrophages are abundant in the human gastrointestinal tract and act key roles in pathogen clearance, regulation of inflammatory response and insulin sensitivity and maintenance of homeostasis.^53^, ^54^ When activated by the external stimuli, macrophages recruit monocytes from the circulatory system to the tumour site and polarise them into TAMs. TAMs interplay with tumour cells via secretions of exosomes or cytokine to facilitate the proliferative, invasive, migratory abilities and angiogenesis of tumour cells. Moreover, TAMs trigger regulatory T cells by the secretion of chemokine, inhibiting the anti‐tumour response of T cells, disrupting the interaction of immune cells and eventually leading to the immune evasion of GC tumour cells. Additionally, TAMs also play a role in GC through metabolic reprogramming and interaction with the microbes (Figure 2). These different mechanisms are detailed in the next section.

### 4.1 Regulation of the phenotypic polarisation of TAMs in GC

**FIGURE 3:**
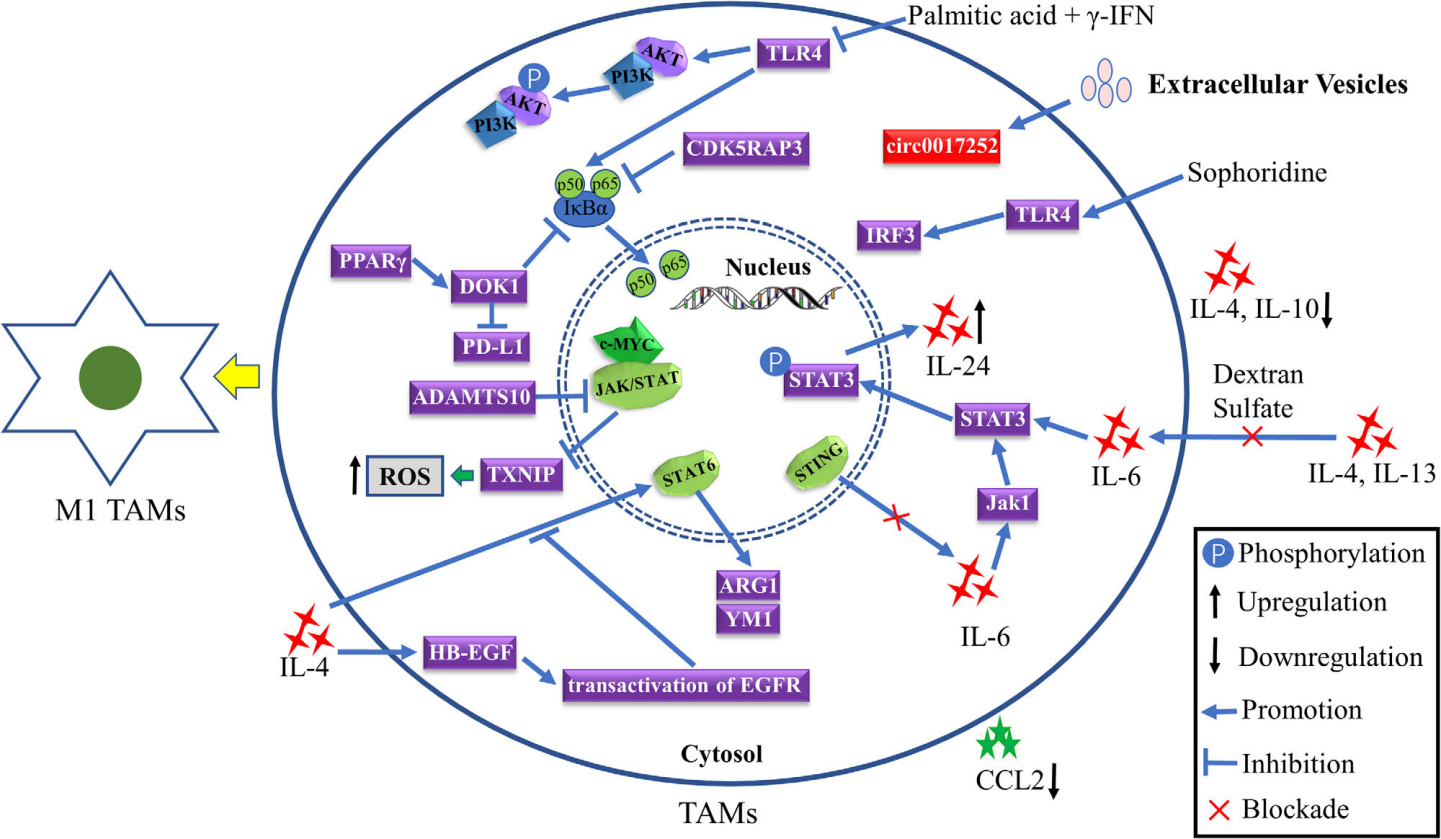
The regulatory mechanisms of tumour‐associated macrophages (TAMs) polarised into classically activated M1 (pro‐inflammatory) macrophages in gastric cancer. IFN, interferon; IL, interleukin.

As tumour initiation usually begins in the chronic inflammatory environment of the epithelial stroma, activated macrophages are dominant in the early stage of tumourigenesis, triggering oncogenes in the adjacent epithelium by producing reactive oxygen species and nitrogen species with other immune cells.^55^, ^56^ After tumourigenesis, more circulating monocytes are recruited to the tumour site and promote tumour progression by secreting various chemokines such as CCL2, CCL5, VEGF and TGF‐β.^57^, ^58^ The inhibition of nuclear factor‐kappa B (NF‐κB) induced the M1 polarisation of TAMs by promoting transcription of pro‐inflammatory cytokines and reducing the secretion of IL‐4 and IL‐10,^33^, ^39^ while activation of NF‐κB enhanced the polarisation of M2 TAMs.^42^ Similarly, targeting STAT pathway decreased the M2 polarisation of TAMs of macrophages,^59^, ^60^ and activating the JAK2/STAT3 pathway facilitated the polarisation of M2 TAMs by increasing the secretion of IL‐6 and IL‐8.^51^, ^61^ As a key communication medium in the TME,^62^ EVs exert a critical function in inducing the polarisation of TAMs.^63^ Accumulative research has reported that EVs containing microRNAs, circular RNAs, long non‐coding RNAs (lncRNAs) or proteins derived from GC cells regulated the polarisation of TAMs after the internalisation by macrophages.^52^, ^64^, ^65^, ^66^, ^67^, ^68^, ^69^, ^70^, ^71^ Notably, natural compounds extracted from plants, such as flavokawain B, sophoridine, diosmetin, elian granules and betulinic acid, were found to enhance the polarisation of M1 TAMs.^72^, ^73^, ^74^, ^75^, ^76^ Additionally, pentraxin‐3 was found to inhibit the polarisation of M2 TAMs by reducing cells secreting IL‐4 and IL‐10 via the inhibition of the phosphorylation of JNK1/2 in GC cells.^77^ IL‐4, a cytokine, was widely reported to elicit M2 polarisation, while Zhao and Liu demonstrated that the IL‐4‐stimulated heparin binding‐epidermal growth factor (HB‐EGF)‐dependent transactivation of EGFR inhibited the polarisation of M2 TAMs induced by IL‐4‐stimulated STAT6 activation.^14^ Furthermore, MMP7 also significantly suppressed the polarisation of M1 TAMs in gastric injury and the development of premalignant lesions.^78^ The regulatory network of phenotypic polarisation of M1 TAMs in GC is presented in Figure 3.

**FIGURE 4:**
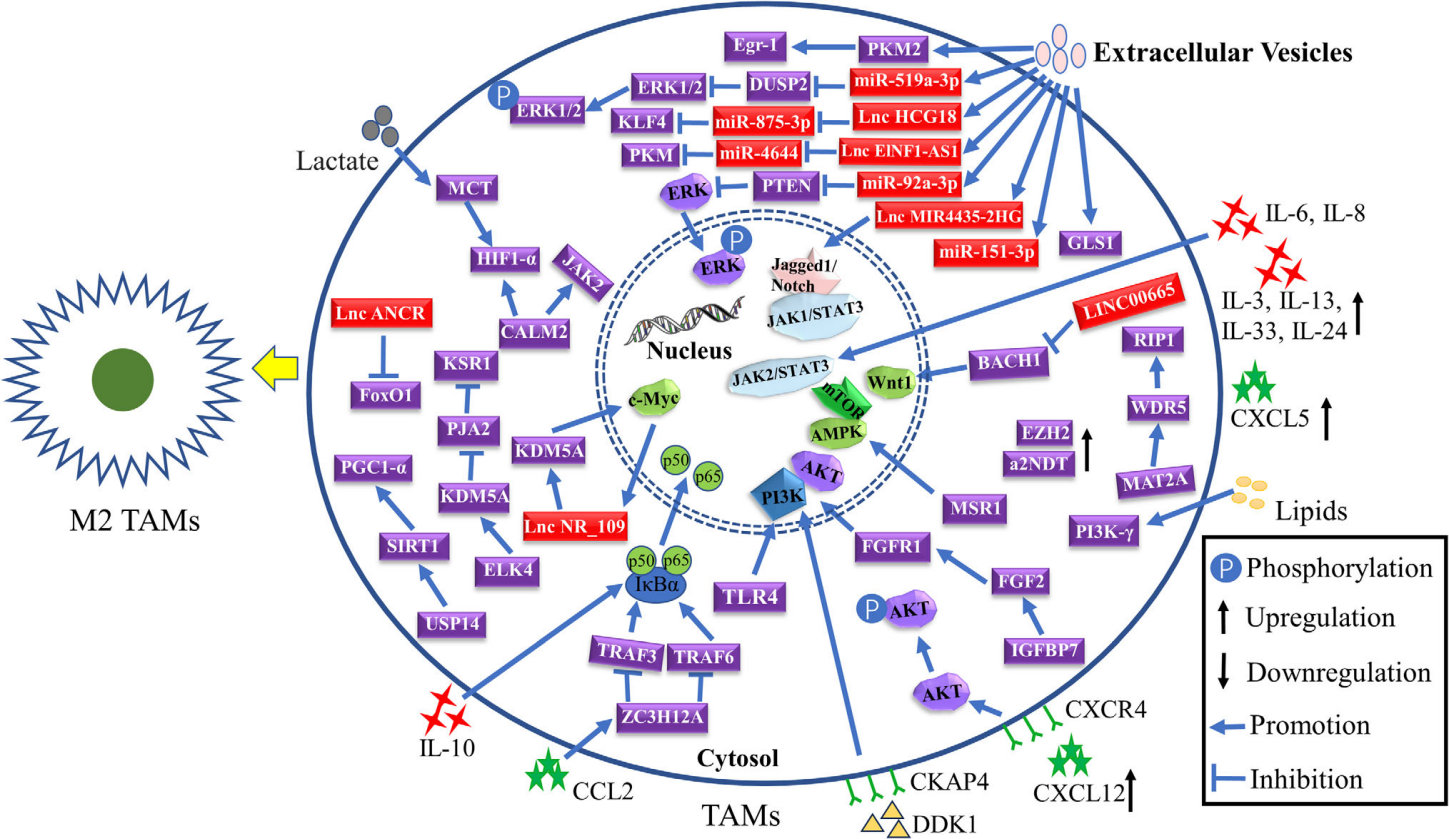
The regulatory mechanisms of tumour‐associated macrophages (TAMs) polarised into classically activated alternatively activated M2 (anti‐inflammatory) macrophages in gastric cancer. IL, interleukin.

TAMs secrete a variety of cytokines and activate multiple signalling pathways to promote cell growth and advanced GC development. Currently, TAMs in the TME are speculated to be mostly induced into cancer‐promoting M2 phenotypes via an anti‐inflammatory and tumour‐promoting activity. The ubiquitin–proteasome system is an important protein regulatory system in eukaryotic cells that is involved in regulating cell cycle transition, cell proliferation and differentiation, signal transduction and other physiological processes.^79^, ^80^ USP14, a deubiquitinase, stabilises SIRT1 via deubiquitination, thereby activating PGC1‐α‐induced M2 polarisation in macrophages.^45^ Several studies have reported that the activation of PI3K/AKT pathway in macrophages induced the M2 TAM polarisation.^48^, ^81^, ^82^, ^83^ Shu et al. revealed that the activation of GPR35 by ERR can enhance the ability of GC cells to induce the polarisation of M2 TAMs.^84^ Similarly, MSR1 facilitated the polarisation of M2 TAMs by enhancing arginine and proline metabolism in macrophages.^46^ Meanwhile, increased levels of chemokines in the TME, CXCL5, CXCL12 and CCL5, can induce a higher proportion of M2 TAMs.^41^, ^83^, ^85^ The results of flow cytometry and immunohistochemistry of a transplanted tumour model indicated that EZH2 induced the polarisation of M2 TAMs.^86^ Furthermore, CCL2 triggered the transcription of ZC3H12A, which upregulated K63‐linked deubiquitination and K48‐linked auto‐ubiquitination of TRAF6/3 to inhibit NF‐κB signalling, thereby reducing M1 TAMs in the TME of HER2‐positive GC.^16^ Also, the non‐coding RNAs (lncRNA ANCR, LINC00665 and lncRNA NR_109) were reported to enhance the polarisation of M2 TAMs in GC.^87^, ^88^, ^89^ Calmodulin 2 was reported to induce the polarisation of M2 TAMs by activating JAK2 or HIF‐1 in macrophages.^90^ Additionally, upregulating IL‐3, IL‐24 and IL‐33 expression promoted the polarisation of M2 TAMs, thereby playing key roles in GC.^32^, ^43^, ^91^ ELK4 inhibited the expression of PJA2 by activating KDM5A to remove H3K4me3 in the PJA2 promoter region, thereby reducing the ubiquitination of KSR1 and stabilising its expression and ultimately promoting M2 polarisation.^92^ Moreover, overexpressed MAT2A elevated the expression of RIP1 by increasing the H3K4 methylation at its promoter regions to induce the polarisation of M2 TAMs.^47^ Several studies have demonstrated that the high expression of TUBA1A, ELK3, GKN2, COL1A2, RAI14, KIF23, GHRL, KCND2, SERPINE1, COL5A2, ALDOC and SLC38A2 and low expression of CD155 and AKR1B10 in GC indicate higher M2 TAM infiltration in the TME using data from public databases.^93^, ^94^, ^95^, ^96^, ^97^, ^98^, ^99^, ^100^, ^101^, ^102^, ^103^, ^104^, ^105^, ^106^, ^107^ Furthermore, TAMs elevated the expression of PI3K‐γ by increasing the uptake of extracellular lipids, thereby inducing M2 phenotype polarisation.^44^ GC cells are characterised by enhanced glycolysis, producing large amounts of lactic acid that can induce the polarisation of M2 TAMs by activating the monocarboxylate channel transporter‐hypoxia inducible factor 1 subunit alpha (MCT–HIF1α) axis in macrophages.^108^ Similarly, the N‐terminal domain of the a2 isoform of vacuolar ATPase was reported to synergistically induce the polarisation of TAMs with M‐CSF.^109^ Mouse forestomach carcinoma cells induced the polarisation of M2 TAMs by increasing the secretion of TGF‐β1.^110^, ^111^ The specific regulatory network of polarisation of M2 TAMs in GC is displayed in Figure 4. Furthermore, the regulatory mechanisms of phenotypic polarisation of TAMs in GC are summarised in Table 1.

**TABLE 1:** The regulation mechanisms of phenotypic polarisation of tumour‐associated macrophages (TAMs) in gastric cancer (GC).

Objects	Expression	Sources	Mechanisms	Outcome	References
EGFR transactivation	–	Macrophages	IL‐4‐stimulated HB‐EGF‐dependent transactivation of EGFR inhibited the polarisation of M2 TAMs induced by IL‐4‐stimulated STAT6 activation.	EGFR transactivation promoted M1 TAMs polarisation	^14^
STING pathway	High expression in GC tissues (200 paired) and macrophages	Macrophages	Knocking‐down STING activated the IL‐6R–JAK–STAT pathway	Knocking‐down STING promoted M1 TAMs polarisation	^32^
DOK1	Low expression in GC tissues (64) and high expression in M1 TAMs (56)	Macrophages	DOK1 activated the NF‐κB pathway	DOK1 promoted M1 TAMs polarisation	^33^
CDK5RAP3	Low expression in GC tissues (241) with high M2 TAMs content	GC cells	CDK5RAP3 inhibited the IL‐4 and IL‐10 by repressing the NF‐κB pathway	CDK5RAP3 promoted M1 TAMs polarisation	^39^
CXCL5	High expression in GC tissues (103) with high M2 TAMs content	Macrophages	–	CXCL5 promoted M2 TAMs polarisation	^41^
CXCL8	High expression in GC tissues (8 paired)	Macrophages	CXCL8 enhanced the secretions of IL‐10 by activating the JAK–STAT1 pathway of GC cells	CXCL8 promoted M2 TAMs polarisation	^42^
YAP1	High expression in GC tissues (32 paired)	GC cells	YAP1 enhanced the secretions of IL‐3 in GC cells	YAP1 promoted M2 TAMs polarisation	^43^
Lipids	–	GC cells	Lipid accumulation activated the PI3K‐γ pathway	Lipids promoted M2 TAMs polarisation	^44^
USP14	High expression in GC tissues (20 paired)	Macrophages	USP14 enhanced the fatty acid oxidation by stabilising SIRT1/PGC1‐α axis	USP14 promoted M2 TAMs polarisation	^45^
MSR1	High expression in GC tissues (21 paired)	Macrophages	MSR1 activated the AMPK/mTOR pathway	MSR1 promoted M2 TAMs polarisation	^46^
MAT2A	High expression in CD14^+^ cells from tumour tissues compared with that from peripheral blood of GC patients (15)	Macrophages	MAT2A enhanced the RIP1 expression by increasing WDR5	MAT2A promoted M2 TAMs polarisation	^47^
IGFBP7	–	CAFs	IGFBP7 activated FGFR1/PI3K/AKT axis through increasing secretions of FGF2	IGFBP7 promoted M2 TAMs polarisation	^48^
IL‐6/IL‐8	–	MSCs	IL‐6 and IL‐8 activated the JAK2/STAT3 pathway	IL‐6 and IL‐8 promoted M2 TAMs polarisation	^51^, ^61^
Exosomal miR‐519a‐3p	High expression in cells with high liver metastasis potential and in serum from GC patients with liver metastasis (75)	GC cells	Exosomal miR‐519a‐3p activated the MAPK/ERK pathway by targeting DUSP2	Exosomal miR‐519a‐3p promoted M2 TAMs polarisation	^52^
ADAMTS10	Low expression in GC tissues (64 paired)	GC cells	ADAMTS10 elevated ROS levels by increasing TXNIP expression through inhibiting JAK/STAT/c‐MYC pathway	ADAMTS10 promoted M1 TAMs polarisation	^59^
Dextran sulphate	–	–	Dextran sulphate blocked the IL‐6–STAT3 pathway induced by IL‐4 and IL‐13	Dextran sulphate promoted M1 TAMs polarisation	^60^
Exosomal circ_0017252	Low expression in GC tissues and serum (5 paired)	GC cells	–	Exosomal circ_0017252 promoted M1 TAMs polarisation	^64^
Exosomal lnc‐MIR4435‐2HG	–	GC cells	Exosomal lnc‐MIR4435‐2HG activated the Jagged1/Notch and JAK1/STAT3 axes	Exosomal lnc‐MIR4435‐2HG promoted M2 TAMs polarisation	^65^
Exosomal PKM2	–	GC cells	Exosomal PKM2 increased the Egr‐1	Exosomal PKM2 promoted M2 TAMs polarisation	^66^
Exosomal miR‐151‐3p	High expression in GC tissues (35)	GC cells	–	Exosomal miR‐151‐3p promoted M2 TAMs polarisation	^67^
Exosomal lnc‐HCG18	High expression in GC tissues (28 paired)	GC cells	Exosomal lnc‐HCG18 increased the KLF4 by targeting miR‐875‐3p	Exosomal lnc‐HCG18 promoted M2 TAMs polarisation	^68^
Sophoridine	–	–	Sophoridine activated the TLR4/IRF3 axis	Sophoridine promoted M1 TAMs polarisation	^73^
PTX3	Low expression in GC tissues (30 paired)	GC cells	PTX3 reduced the secretions of IL‐4 and IL‐10 from GC cells by inhibiting phosphorylation of the JNK1/2	PTX3 promoted M1 TAMs polarisation	^77^
DKK1	High expression in GC tissues (284 tumour samples and 32 paired normal tissues)	GC cells	DKK1 interacted with CKAP4 on the macrophage surface and activated downstream PI3K–AKT signalling	DKK1 promoted M2 TAMs polarisation	^81^
POU1F1	High expression in GC tissues (60 paired)	GC cells	HMGA1B/2 triggered CXCL12/CXCR4 axis by transcriptionally activating POU1F1	POU1F1 promoted M2 TAMs polarisation	^83^
CRIP1	High expression in GC tissues (81 paired)	GC cells	CRIP1 increased the CCL5 secretion by promoting phosphorylation of CREB1	CRIP1 promoted M2 TAMs polarisation	^85^
LINC00665	High expression in M2 TAMs	Macrophages	LINC00665 activated the Wnt1 by binding to BACH1	LINC00665 promoted M2 TAMs polarisation	^87^
LncRNA ANCR	High expression in GC tissues (60)	Macrophages	LncRNA ANCR inhibited the expression of FoxO1	LncRNA ANCR promoted M2 TAMs polarisation	^88^
CALM2	High expression in GC tissues (31 paired) and cell lines	GC cells	CALM2 increased the CXCL12, IL‐4, IL‐13 and IL‐10 by activating JAK2/STAT3/HIF‐1 signalling	CALM2 promoted M2 TAMs polarisation	^90^
ELK4	High expression in GC tissues (30 paired) and GC‐TAMs	Macrophages	ELK4 increased the KSR1 by regulating the KDM5A–PJA2 axis	ELK4 promoted M2 TAMs polarisation	^92^
Lactic acid	–	GC cells	Lactic acid triggered MCT–HIF1α signalling	Lactic acid promoted M2 TAMs polarisation	^108^

Abbreviations: AMPK/mTOR, AMP‐activated protein kinase/mammalian target of rapamycin; CAFs, cancer‐associated fibroblasts; CCL, C‐C motif chemokine ligand; HB‐EGF, heparin binding‐epidermal growth factor; IL, interleukin; MCT–HIF1α, monocarboxylate channel transporter‐hypoxia inducible factor 1 subunit alpha; MSCs, mesenchymal stromal cells; NF‐κB, nuclear factor‐kappa B; PI3K/Akt, phosphatidylinositol‐3‐kinase/protein kinase B; ROS, reactive oxygen species.

### 4.2 TAMs regulate tumour proliferation, invasion and migration

In the TME, TAMs and tumour cells interact through mediators such as cytokines to facilitate cell growth. The migrative and invasive abilities of the cell are reported to be enhanced by M2 TAMs via increasing the secretion of MMP2 and promoting the epithelial–mesenchymal transition in GC cells.^39^ Meanwhile, Zhang et al. revealed that the mixed TAMs of M1 and M2 promoted the malignant biological behaviours of diffuse‐type GC by increasing the CAFs via the secretion of IL‐1β.^50^ Furthermore, TAMs under hypoxic conditions activated CXCR1/2 to boost the cell growth and metastatic behaviours of GC in vitro and in vivo by increasing CXCL8 secretion.^42^ Miao et al. demonstrated that the inhibition of the STING pathway of TAMs promoted apoptotic effects on GC cells.^32^ The migrative and invasive abilities of GC cells were enhanced by M2 TAMs via the increased activation of COX2/MMP9.^40^ Additionally, TAMs activated the TNFR1–ERK–VGLL1 signalling to increase the cell viability of GC by increasing TNF‐α secretion.^112^ Furthermore, EVs containing ApoE derived from M2 TAMs activated the PI3K–Akt pathway to enhance the cell migration of GC cells.^38^ Several studies have reported and validated the promoting effects of M2 TAMs on cell migration and invasion in GC.^14^, ^60^, ^61^, ^85^, ^88^, ^109^, ^113^, ^114^, ^115^, ^116^

### 4.3 The role of TAMs in the chemoresistance of GC

Due to the atypical early symptoms of GC, more than 80% of hospitalised patients are initially diagnosed with locally advanced or metastatic GC.^117^, ^118^ This phenomenon warranted neoadjuvant therapy and postoperative adjuvant chemotherapy for GC treatment. Therefore, uncovering the mechanism of drug resistance and increasing the sensitivity of chemotherapy drugs will be of great benefit to improve the overall survival (OS) of patients with GC. TAMs were reported to decrease the sensitivity of GC cells to oxaliplatin and doxorubicin by producing the EVs containing circular RNA 0008253^37^ and miR‐223.^119^ Also, M2 TAMs activated the PI3K/AKT/mTOR pathway to confer resistance to 5‐fluorouracil (5‐FU) by improving the level of CXCL5 in GC cells.^41^ Several studies observed that decreased M1 TAMs or increased M1 TAMs conferred trastuzumab resistance in HER2‐positive GC cells.^16^, ^71^ Additionally, the transfer of EVs containing lncRNA CRNDE derived from M2 TAMs to GC cells conferred cisplatin resistance by promoting neural precursor cell expressed developmentally downregulated protein 4‐1 (NEDD4‐1)‐mediated ubiquitination and degradation of phosphatase and tensin homolog (PTEN).^36^ Similarly, the transfer of EVs containing miR‐588 derived from M2 TAMs to GC cells also conferred cisplatin resistance by targeting CYLD.^35^ Moreover, M2 TAMs activated the JAK1/STAT3 pathway and conferred resistance to 5‐FU by increasing the secretion of CCL8 in tumour cells.^43^ Furthermore, Ngabire et al. revealed that M2 TAMs elevated the abundance of Integrin β3, FAK and Cofilin to confer the resistance of GC cells to 5‐FU.^120^ Meanwhile, the transfer of EVs containing miR‐21 derived from M2 TAMs to GC cells conferred cisplatin resistance by activating the PI3K/AKT pathway and reducing PTEN levels.^34^

### 4.4 TAMs enhance angiogenesis in GC

As one of the 14 hallmarks of tumour development, vasculature induction plays key roles in tumour progression.^121^ Also, the functions of TAMs in promoting GC angiogenesis cannot be ignored.^122^, ^123^ Qiu et al. reported that M2 TAMs enhanced the liver metastasis of GC by promoting the formation of the microenvironment before intrahepatic metastasis and the development of angiogenesis after metastasis.^52^ Guo et al. validated the promoting effects on the angiogenesis of M2 TAMs in GC.^60^ Moreover, the promoting effects of M2 TAMs on angiogenesis in GC were confirmed by Tang et al.^83^ Notably, high microvessel density (CD105) was observed in the tissues with high infiltration of M2 TAMs using immunohistochemical (IHC) staining.^124^

### 4.5 TAMs regulate immunity in the TME of GC

As an important effector cell of the immune system, macrophages play a key regulatory role in the immune evasion of tumour cells by producing various chemokines and cytokines.^125^, ^126^ Using the IHC analyses of 448 GC samples, He et al. revealed that an increased number of basophils tended to have more M2 TAM infiltration, which was closely related to the immune evasion environment and indicated a worse prognosis.^127^ Shi et al. found that CD47 expression was positively correlated with M1 TAM infiltration using the IHC analysis of 453 GC samples.^128^ Meanwhile, M2 TAMs increased lipid levels, resulting in decreased phagocytic capacity and increased PD‐L1 expression that blocked T‐cell‐mediated immune killing, thereby inhibiting the immune response of the TME in GC tumours.^44^ The inhibition of the STING pathway in TAMs upregulated the tumour‐infiltrating CD8^+^ T cells and CD8^+^/CD4^+^ ratio of tumours in vivo.^32^ Zhang and coworkers demonstrated that the sophoridine‐induced polarisation of M1 TAMs could increase the proliferation and killing functions of CD8^+^ T cells by increasing the expression of Granzyme‐B, TNF‐α and perforin in vitro.^73^ Similarly, Huang et al. revealed that PD‐L1 was increased in CD68‐only and CD206+ TAMs.^129^ Furthermore, the polarisation of ILC2s in the peripheral blood could lead to the formation of an immunosuppressive microenvironment in patients with GC by upregulating M2 TAMs.^130^ Guo et al. reported that high infiltration of SIGLEC10‐positive TAMs usually indicated low levels of CD8^+^ T‐cell infiltration in TME.^131^

### 4.6 Metabolic alterations of TAMs in GC

The metabolic pathways of M1 and M2 macrophages are different. Alterations in metabolic enzymes, metabolites and metabolic pathways in macrophages profoundly affect the tumour progression of GC.^66^, ^132^, ^133^ M2 TAMs enhance the malignant biological behaviours of GC cells by increasing the oxidation of fatty acids.^45^ Additionally, M2 TAMs activate the AMP‐activated protein kinase/mammalian target of rapamycin (AMPK/mTOR) pathway to enhance autophagy signalling in GC cells by increasing arginine and proline metabolism.^46^ The GLUT3‐dependent glycolysis metabolism of TAMs has been reported to confer resistance to 5‐FU by increasing the secretion of CCL8 in tumour cells.^43^ Furthermore, M2 TAMs aggravate GC progression by increasing the methionine metabolism^47^ and tumour progression by increasing lipid levels to repress the immune response of the TME in GC cells.^44^

### 4.7 Interaction between TAMs and the microbes in GC

Increasing evidence shows that the microbiota in the stomach affects the occurrence and development of GC directly or indirectly via its immunomodulatory activities.^134^, ^135^ TAMs, which are important immune cells in the TME of GC, have been reported to naturally interact with the microbiota in the stomach. Li et al. reported that Propionibacterium acnes activated the TLR4/PI3K/Akt pathway to enhance the differentiation of M2 TAMs, thus boosting the migratory ability of GC cells.^82^ Also, *Lactobacillus gasseri* Kx110A1 was found to inhibit the secretion of TNF and IL‐6 in host macrophages via *H. pylori* infection by decreasing the expression of ADAM17.^136^ Similarly, Suarez et al. reported that macrophages shifted significantly from M2 to M1 type after *H. pylori* infections in animal models of GC.^137^ The mannose‐sensitive hemagglutination pilus strain of *Pseudomonas aeruginosa*, a genetically engineered bacterium, was reported to effectively induce the polarisation of M1 TAMs by activating the NF‐κB pathway.^138^ Furthermore, MMP7 inhibited the development of *H. pylori*‐induced gastric injury and precancerous lesions by reducing the polarisation of M1 TAMs.^78^

## 5 APPLICATIONS OF TAMS IN GC TREATMENT

### 5.1 Inhibition of M2 TAM infiltration in GC

Inhibiting the infiltration of M2 TAMs in the TME is a valuable exploration for the advancement of GC therapy. He et al. reported that IU1 (a specific inhibitor of USP14) effectively reduced the proportion of M2 TAMs in the TME in vivo.^45^ Notably, modified Jianpi Yangzheng, a traditional Chinese medicine, was found to effectively inhibit the differentiation of M2 TAMs by reducing the content of PKM in the EVs of GC.^66^ Similarly, the conditioned media from Flavokawain B (extracted from the rhizomes of *Alpinia pricei* Hayata)‐treated GC cells was reported to reduce the polarisation of M2 TAMs.^72^

### 5.2 Repolarisation of TAMs

The plasticity of TAMs allows researchers to induce the conversion of the M2 into M1 type, which is a potential target in GC treatment strategies. As shown in Table 2, researchers have started to utilise the plasticity or unique surface markers of TAMs to explore a feasible targeted strategy in order to be able to translate into clinical therapy. αVEGFR2–MICA fusion antibodies were found to convert M2 into M1 TAMs in vitro and in vivo by targeting the VEGFR2 and MICAα1–α2 ectodomain of macrophages in GC.^15^ Li et al. demonstrated that PPARγ‐agonists enhanced the polarisation of M1 TAMs by elevating the expression of DOK1.^33^ In addition, PI3K‐γ inhibitor (IPI‐549) was found to reverse the inhibition of phagocytosis and polarisation of M2 TAMs that induced by lipid accumulation and enhanced anti‐tumour activity.^44^ Similarly, USP14 inhibitor (IU1) can effectively repolarise TAMs from M2 to M1 and enhance anti‐tumour activity in vivo.^45^ In addition to repolarising TAMs from M2 to M1 by activating the TLR4–IRF3 axis, sophoridine has been demonstrated to promote the growth and cytotoxic effects of CD8^+^ T cells while alleviating their exhaustion.^73^ Moreover, umbelliprenin, a sesquiterpene coumarin, increased the M1/M2 ratio of TAMs and IL‐12 and nitric oxide (NO) levels in the supernatant of M1 TAMs but decreased IL‐10 and IL‐12 levels in the supernatant of M2 TAMs.^139^ Notably, Jianpi Yangzheng Decoction, a traditional Chinese medicine, was reported to effectively repolarise TAMs from the M2 to M1 type.^140^ Surprisingly, Chen et al. reported that carboxymethylated alginate‐resiquimod micelles can effectively repolarise TAMs from M2 to M1 type in vivo, thereby enhancing the anti‐tumour effects of chemotherapy and immunotherapy and prolonging the survival time of mice.^141^ Surprisingly, three clinical trials are currently conducted worldwide by utilising the plasticity or unique surface markers of TAMs (Table 3). An interventional clinical trial (phase 1, NCT04660929) led by the Heidelberg National Cancer Center in Germany is underway to recruit patients with HER2‐overexpressing solid tumours, including GC. CAR‐macrophages achieves the therapeutic efficacy by modifying autologous macrophages containing anti‐HER2 chimeric antigen receptors (CT‐0508). The ability of macrophages to infiltrate tumours, phagocytosis and antigen presentation makes CAR‐macrophages a potent target. Dong et al. reported that CAR‐macrophages (expressing HER2‐FcεR1γ‐CAR) alone or in combination with oxaliplatin can boost the anti‐tumour effects on HER2‐positive GC in vitro and in vivo.^142^ The CSF‐1/CSF‐1R axis has always been one of the focuses for targeting TAMs.^143^, ^144^, ^145^ An interventional clinical trial (phase 2, NCT03694977) led by Seoul National University Hospital in Korea was conducted to explore the therapeutic efficacy of CSF‐1 inhibitor (MCS110) combined with PD‐1 inhibitor (PDR001) in patients with advanced GC. CD47 was elevated widely across tumour types and exerted a critical role in inhibiting cell performance through binding to the transmembrane protein SIRPα in phagocytic cells.^146^, ^147^, ^148^, ^149^ In addition, CD47 was positively correlated with TAMs infiltration using the IHC analysis in 453 GC samples.^128^ An interventional clinical trial (phase 1, NCT05482893) led by MD Anderson Cancer Center is conducted to explore the therapeutic efficacy of Claudin 18.2 and CD47 inhibitor (PT886) in patients with advanced gastric and gastroesophageal junction adenocarcinomas. Abovementioned clinical trials are unveiling the prelude to the clinical translational therapy of TAMs as therapeutic targets.

**TABLE 2:** Therapeutic strategy targeting tumour‐associated macrophages (TAMs) in gastric cancer (GC).

Strategy	Mechanisms	In vitro/in vivo model	Outcome	References
Repolarising TAMs (from M2 to M1)	αVEGFR2–MICA fusion antibodies	In vitro RAW 264.6, PBMCs, NK‐92 and in vivo BGC‐823 and AGS cell line	Induced the polarisation of RAW264.7 to M1 type and enhanced cellular cytotoxicity and anti‐tumour efficacy	^15^
Targeting TAMs in HER2‐positive GC	CD40×HER2 bispecific antibody, which targeted the CD40 to restore the ubiquitination level of TRAF6/3 and activate NF‐κB signalling, increased the M1 TAMs	In vitro RAW 264.6 and THP‐1 cell line and in vivo NCI‐N87 cell line	CD40×HER2 bispecific antibody increased M1 TAMs, enhanced anti‐tumour activity and overcome trastuzumab resistance in HER2‐positive GC	^16^
Enhancing the polarisation of M1 TAMs	PPARγ‐agonists (rosi) activated the NF‐κB pathway by elevating the expression of DOK1	In vitro THP‐1 and AGS cell line	Elevated M1 TAMs populations and enhanced cytotoxic efficacy of macrophages towards AGS cells	^33^
Repolarising TAMs (from M2 to M1)	PI3K‐γ inhibitor (IPI‐549)	In vitro BMDMs and in vivo MFC cell line	IPI‐549 reversed the inhibition of phagocytosis, polarisation of M2 TAMs and enhanced anti‐tumour activity	^44^
Repolarising TAMs (from M2 to M1)	USP14 inhibitor (IU1) blocked the SIRT1/PGC1‐α axis	In vivo MFC cell line	IU1 repolarised TAMs from M2 to M1 and enhanced anti‐tumour activity	^45^
Repolarising TAMs (from M2 to M1)	Dextran sulphate blocked the IL‐6–STAT3 pathway induced by IL‐4 and IL‐13	In vivo BGC‐823	Dextran sulphate reduced the infiltration of M2 TAMs in intraperitoneal metastatic tumours	^60^
Repolarising TAMs (from M2 to M1)	Sophoridine activated the TLR4/IRF3 axis	In vitro RAW 264.6 and THP‐1 cell line and murine CD8^+^ T cells	Sophoridine repolarised TAMs from M2 to M1 and stimulated the proliferation and cytotoxic function of CD8^+^ T cells, and relieved CD8^+^ T‐cell exhaustion	^73^
Repolarising TAMs (from M2 to M1)	DKK1 antibody inactivated PI3K–AKT signalling by targeting DKK1	In vitro BMDMs, CD8^+^ T cells and MFC cell line and in vivo MFC cell line	DKK1 antibody repolarised TAMs from M2 to M1 and boosted the tumour‐killing function of CD8^+^ T cells and the efficacy of PD‐1 inhibitors	^81^
Targeting SIGLEC10‐positive TAMs	SIGLEC10 antibody	In vitro tumour single‐cell suspensions	SIGLEC10 antibody enhanced the tumour‐killing function of CD8^+^ T cells	^131^
Repolarising TAMs (from M2 to M1)	Umbelliprenin decreased IL‐10 and increased IL‐12 and NO	In vitro THP‐1 cell line	Umbelliprenin significantly increased the M1/M2 ratio	^139^
Repolarising TAMs (from M2 to M1)	–	In vitro THP‐1 cell line	Jianpi Yangzheng Xiaozheng Decoction repolarised TAMs from M2 to M1	^140^
Repolarising TAMs (from M2 to M1)	HSA‐Au (III) α‐N‐heterocyclic thiosemicarbazone compounds (5b) nanoparticles promoted the NF‐κB and iNOS and inhibited Msr2 and STAT3	In vitro RAW264.7 cell line and in vivo MFC cell line	HSA‐5b nanoparticles promoted the polarisation of M1 TAMs and enhanced anti‐tumour activity and recruitment of CD4^+^ T cells, CD8^+^ T cells and NK cells in the tumours	^154^
Enhancing the polarisation of M1 TAMs	The immunonutrition activated the inflammatory pathway in TME	*–*	The immunonutrition increased the M1 TAMs infiltration and decreased the M2 TAMs infiltration in TME	^155^
Targeting M2 TAMs	A mannose‐conjugated chlorin (M‐chlorin) was designed to bind to the mannose receptor, which is highly expressed on M2 TAMs	In vitro THP‐1 cell line	Photodynamic therapy with M‐chlorin effectively induced M2 TAMs death	^156^
Targeting CD47‐positive TAMs	CD47 antibody	In vitro THP‐1 cell line and in vivo MFC cell line	CD47 antibody enhanced phagocytosis and IFN‐β secretions of TAMs in Epstein–Barr virus‐associated GC	^157^
Targeting C5aR1‐positive TAMs	C5aR1 antibody	In vitro tumour single‐cell suspensions	C5aR1 antibody promoted the secretion of pro‐inflammatory cytokines TNF‐α and IL‐1β by Dectin‐1+ TAM and boosted the tumour‐killing function of CD8^+^ T cells and the efficacy of PD‐1 inhibitors	^158^
Targeting Dectin‐1‐positive TAMs	Dectin‐1 antibody	In vitro tumour single‐cell suspensions	Dectin‐1 antibody promoted the secretion of pro‐inflammatory cytokines TNF‐α and IL‐1β by Dectin‐1+ TAM and boosted the tumour‐killing function of CD8^+^ T cells and the efficacy of PD‐1 inhibitors	^159^

Abbreviations: BMDMs, bone marrow‐derived macrophages; HSA, human serum albumin; IL, interleukin; NF‐κB, nuclear factor‐kappa B; PBMCs, peripheral blood mononuclear cells; TME, tumour microenvironment; TNF‐α, tumour necrosis factor‐α.

**TABLE 3:** Clinical trials of macrophage repolarization immunotherapy in gastric cancer.

Target	Class	Compound	Combination/monotherapy	Clinical phase/study type	Sponsors/collaborators	ClinicalTrials.gov ID no.	Status
HER‐2 overexpressing tumours	CAR‐macrophages	CT‐0508	Monotherapy	Phase 1/interventional	National Center for Tumour Diseases, Heidelberg/University Hospital Heidelberg Carisma Therapeutics Inc.	NCT04660929	Recruiting
CSF‐1/CSF‐1R axis	CSF‐1 inhibitors	MCS110	MCS110/PDR001 combination	Phase 2/interventional	Seoul National University Hospital	NCT03694977	Recruiting
Claudin 18.2 and CD47	Claudin 18.2 and CD47 inhibitors	PT886	Monotherapy	Phase 1/interventional	MD Anderson Cancer Center/Phanes Therapeutics	NCT05482893	Recruiting

### 5.3 Targeting TAMs in immunotherapy

Immunotherapy is the first‐line treatment for comprehensive therapy of patients with advanced GC, greatly prolonging the OS of patients.^150^, ^151^, ^152^ Immune checkpoint inhibitors (ICIs) are commonly the main drugs for GC immunotherapy and include antibodies targeting CTLA‐4, PD‐1 and PD‐L1. Therefore, the exploration of the combined targeting of TAMs and ICIs in immunotherapy has promising therapeutic potential. Pan et al. revealed that αVEGFR2–MICA fusion antibodies combined with anti‐PD‐1 antibodies can enhance the efficacy of immunotherapy by reprogramming the polarisation of TAMs in GC.^15^ Additionally, Smith et al. reported that gastrin vaccine alone or combined with PD‐1 antibody restrained GC growth and metastasis by reducing the M2 TAMs in the TME.^153^ Researchers used CD40–HER2 bispecific antibody to target CD40 and maintain the ubiquitination level of TRAF6/3, thereby increasing its degradation in macrophages and consequently activating the NF‐κB pathway to increase M1 TAMs and offset trastuzumab resistance in HER2‐positive GC cells.^16^ Furthermore, Guo et al. reported that dextran sulphate reduced the promoting effects of M2 TAMs on the angiogenesis, and migratory and invasive abilities of GC cells using co‐culture assays.^60^ Shi et al. revealed that targeting DKK1 can effectively repolarise TAMs from M2 to M1 and augment the tumour‐killing function of CD8^+^ T cells and the efficacy of PD‐1 inhibitors by blocking PI3K/AKT signalling of macrophages in vitro and in vivo.^81^ Moreover, combining an Au agent for chemotherapy and immunotherapy obtained satisfactory tumour‐killing results by targeting TAMs in the TME in vitro and in vivo.^154^ Furthermore, preoperative oral immunonutrition in patients with GC increased M1 TAM infiltration in the TME.^155^ A mannose‐conjugated chlorin (M‐chlorin) was designed to bind to the mannose receptor, which is highly expressed on M2 TAMs, and photodynamic therapy with M‐chlorin efficiently induced M2 TAMs apoptosis and enhanced the killing effects on GC tumours.^156^ Duan et al. demonstrated that targeting CD47 was a potent immunotherapy by enhancing phagocytosis and IFN‐β secretions of TAMs through activating cGAS‐STING signalling in Epstein–Barr virus‐associated GC.^157^ When targeting SIGLEC10,^131^ C5aR1^158^ and Dectin‐1^159^ mainly expressed on TAMs not only reprogrammed TAMs but also boosted the tumour‐killing function of CD8^+^ T cells and the efficacy of PD‐1 inhibitors. Melatonin, one of the autocrine hormones in the human body, has been reported to reduce the PD‐L1 expression in TAMs and enhance the function of CD8^+^ T cells by affecting the EVs secretions of GC cells.^160^ Abovementioned facts indicate that targeting TAMs alone or in combination with ICIs would be a promising therapy for GC, particularly for advanced patients.

## 6 THE RELATIONSHIP BETWEEN TAMS AND THE PROGNOSIS OF PATIENTS WITH GC

The role of TAMs in the progression of GC is complex and dynamic owing to their varying tumour‐suppressing or tumour‐promoting activity that is dependent on time and space. Thus, different types of TAMs usually indicate different prognoses and therapeutic responses in patients with GC (Table 4).^161^, ^162^ Li et al. reported that high infiltration of M1 TAMs (IL‐18) not only positively correlated with the microsatellite instable/microsatellite stable (MSI/MSS)‐sig but also predicted better OS in GC patients with stage IV (94 samples) by using proteomic analysis.^162^ Su et al. found that patients with high infiltration of macrophages (CD68) were often related to poor response to neoadjuvant chemotherapy in a 103 sample cohort.^41^ Meanwhile, high infiltration of M2 TAMs (CD206) was usually forecasted as resistant to trastuzumab therapy in patients with HER2‐positive GC.^71^ Sun et al. reported that the high expression of total TAMs (CD68) or M2 TAMs (CD206) usually predicted a poor OS, while the high expression of M1 TAMs (CD86) indicated a better prognosis in HER2‐positive patients with GC.^16^ Similarly, in a 433 sample cohort, a high proportion of M1 TAMs (CD11c) predicted a better OS in patients with GC.^128^ Moreover, Tang et al. demonstrated that patients with GC having a high density of M2 TAM (CD163) infiltration indicated a poor OS compared with those having a low density of M2 TAM infiltration.^83^ Meanwhile, the high expression of total TAMs (CD68), M2 TAMs (CD163) or CD47‐positive TAMs predicted worse OS of GC patients was verified in a 423 sample cohort.^157^ Besides, the intestinal‐type patients with GC having a high density of M2 TAMs (IL10, IL13 or CD163) and total TAM (CD68) infiltration usually predicted a worse OS compared with their low infiltration counterparts.^163^ Xu et al. observed that patients with GC having a high infiltration of M2 TAMs (CD204) usually predicted a shorter OS compared with those having a low infiltration of M2 TAMs in a cohort comprising 228 cases.^40^ On investigation of the spatial heterogeneity of TAMs in GC, researchers found that patients with a high density of M2 TAMs within the core were correlated with have better relapse‐free survival (RFS) but not a better OS rate. Additionally, patients with GC having a higher effective density of M2 TAMs (0−10 μm) had significantly longer RFS and OS compared to those with lower TAMs density.^129^ Also, patients with GC having a higher infiltration of total TAMs (CD68) predicted a shorter OS compared to those with a lower infiltration of total TAMs.^113^ In addition, Huang et al. reported that patients with GC having a high infiltration of total TAMs (CD68) or M2 TAMs (CD163) often correlated with short disease‐free survival (DFS) and disease‐specific survival (DSS) rates.^164^ Similarly, Zhang et al. demonstrated that patients with GC having a high infiltration of total TAMs (CD68) or M2 TAMs (CD163) often correlated with worse OS, whereas those (including patients with recurrent GC) with elevated levels of M2 TAMs (CD163) had a worse DSS rate.^165^, ^166^ Park et al. reported that the distribution of TAMs in various histologic locations had different predictive abilities for the prognosis of patients with GC. The patients with GC having a high infiltration of M2 TAMs (CD163) in the tumour stroma and invasive tumour margin often predicted a poor OS, while the distribution of high infiltration of M2 TAMs in the tumour nest was not related to the prognosis of patients.^124^ This phenomenon was significant for TAMs in different histologic locations to predict the prognosis of GC patients more accurately, and more clinical samples were needed to verify this hypothesis. Notably, Kim et al. reported that the higher infiltration of M2 TAMs (CD163) predicted a better DFS in microsatellite instability‐high patients with GC.^167^ Furthermore, Zhang et al. revealed that patients with GC having a higher infiltration of M2 TAMs (CD163) or lower infiltration of M1 TAMs (CD11c) usually had a worse OS.^168^ Additionally, patients with high M1 density or M1/M2 ratio were reported to have a higher OS compared to those with low M1 density or M1/M2 ratio after radical surgery for GC.^169^ In addition to the classic surface markers of TAMs, the clinical predictive value of some relatively specific markers expressed in TAMs has also been explored. High infiltration of SIGLEC10‐positive TAMs often forecasted worse OS and more advanced clinical stage of GC patients (143 samples).^131^ Zhang et al. reported that high expression of C5aR1‐positive TAMs not only forecasted the poor OS (459 samples) for GC patients but also predicted worse response to 5‐FU for advanced GC patients (342 samples).^158^ Similarly, Liu et al. found that high infiltration of Dectin‐1‐positive TAMs usually predicted worse OS and DFS of GC patients (451 samples).^159^

**TABLE 4:** Literature reports on the associations between tumour‐associated macrophages (TAMs) and the prognosis of gastric cancer (GC) patients.

Study result	Marker in TAMs	Sample size (case)	References
The higher expression of CD68 or CD206 predicts poorer survival outcomes in HER2‐positive GC patients, while the higher expression of CD86 indicates a better prognosis	Total TAMs: CD68 M1 TAMs: CD86 M2 TAMs: CD206	33	^16^
Patients with high CD204 TAMs infiltration had a shorter 5‐year survival time of GC	M2 TAMs: CD204	228	^40^
The GC patients with high CD163 expression exhibited poor OS, compared with patients expressing low levels of CD163	M2 TAMs: CD163	60	^83^
GC patients with a higher expression of CD68 had a shorter OS when compared with patients with a lower expression of CD68	Total TAMs: CD68	60	^113^
GC patients with high infiltration of CD163 TAMs in tumour stroma and invasive tumour margin were significantly correlated with poor OS	M2 TAMs: CD163	113	^124^
The high CD11c expression was a prognosticator for better OS in GC patients	M1 TAMs: CD11c	433	^128^
In a univariate analysis of outcome, M2 TAMs within the core were associated with improved RFS, but not OS. GC patients with a higher effective density of M2 TAMs (0−10 μm) had significantly longer RFS and OS when compared with patients with lower TAM density	Total TAMs: CD68^+^ and AE1AE3^−^ M1 TAMs: CD68^+^, CD163^−^ and CD206^−^ M2 TAMs: CD68^+^, CD163^+^ or CD206^+^	51	^129^
GC patients with high infiltration of SIGLEC10‐positive TAMs usually predicted worse OS	SIGLEC10	134	^131^
GC patients with high expression of CD68, CD163 or CD47 predicted worse OS	Total TAMs: CD68 M2 TAMs: CD163	423	^157^
GC patients with high expression of C5aR1‐positive TAMs indicated poor OS and worse response to 5‐fluorouracil	C5aR1	459/342	^158^
GC patients with high infiltration of Dectin‐1‐positive TAMs usually predicted worse OS and DFS	Dectin‐1	451	^159^
High infiltration of M1 TAMs predicted better OS in GC patients with stage IV	M1 TAMs: IL‐18	94	^162^
A low density of CD163 TAMs was significantly associated with poor DFS in microsatellite instability‐high GC patients	Total TAMs: CD68 M2 TAMs: CD163	143	^167^
High versus low CD11c density and low versus high CD206 density indicated better OS in GC patients	Total TAMs: CD68 M1 TAMs: CD11c M2 TAMs: CD206	180	^168^
GC patients with high M1 density or M1/M2 ratio had a higher OS compared with those with low M1 density or M1/M2 ratio	M1 TAMs: CD68 and NOS2 M2 TAMs: CD68 and CD163	52	^169^

Abbreviations: DFS, disease‐free survival; IL, interleukin; OS, overall survival; RFS, relapse‐free survival.

## 7 DISCUSSION

As a malignant gastrointestinal tumour, the incidence and mortality of GC are gradually increasing worldwide. The occurrence and development of GC is a complex, multi‐step and multi‐factor process. The polarisation of TAMs, an important part of the GC TME, is affected by multiple signalling pathways and surrounding cells. This article reviews the phenotypic polarisation, function and molecular mechanism of TAMs and their potential applications in the treatment selection and prognostic prediction of GC. TAMs exert carcinogenic functions by facilitating tumour cell growth, migration and invasion; promoting angiogenesis; inhibiting anti‐tumour immunity; reprogramming metabolic patterns; and interplaying with microorganisms in the stomach. Based on the summary of the role and mechanism of TAMs in GC, the orientations of TAMs as potential therapeutic targets can be via the inhibition of monocyte infiltration, reprogramming of TAMs’ polarisation and use of TAMs as a direct target for immunotherapy. Owing to the diversity and complexity of TAMs, tumour subtypes, clinical staging and challenges in individualised treatments, the exploration of appropriate TAM‐related prognostic markers or a combination of other immune cell indicators poses various challenges.

Currently, the investigation of TAMs in GC remains insufficient. Single‐cell RNA sequencing (scRNA‐seq) eliminates heterogeneity in tissues by clustering cells, which is of great significance for the initiation and development of malignancies.^170^, ^171^, ^172^ Sathe et al. reported that macrophages were heterogeneous at the transcriptional level through scRNA‐seq, which did not conform to the traditional M1/M2 paradigm.^173^ If further studies integrate single‐cell proteomics and transcriptomics analysis, this may provide a more detailed analysis of the subtypes of macrophages in GC TME to better classify macrophages and their corresponding functions in GC. Despite the fact that TAMs are being used as therapeutic targets in three clinical trials for GC, these studies are all still in the early stages of clinical translational research. More clinical trials related to TAMs need to be conducted to investigate the precise treatment of GC patients. Besides, the role of TAMs in GC progression is diverse and changes with the development of tumours. A majority of studies have revealed that the high infiltration of macrophages usually predicted good outcomes for patients; however, there do exist controversial conclusions. The possible reasons for these inconsistent results are summarised as follows: (1) the diversity of markers for identifying TAM phenotypes and the lack of identification criteria; (2) the spatial heterogeneity of GC tumours and the role of TAMs in different locations are diverse; (3) the markers for identifying TAM phenotypes lack specificity.

Recent studies have demonstrated that TAMs in the TME of GC cells can express PD‐1 and PD‐L1. Hence, immunotherapy targeting TAMs could synergistically strengthen the efficacy of ICIs, thereby enhancing patient response to immunotherapy. Additionally, reprogramming the polarisation and function of TAM subtypes at different stages of GC development could be considered a prospective treatment strategy. Although the current clinical trials of TAMs were in the early stage and there is no significant evidence, the abilities of TAMs in TME indicate that they will be a potent target in GC. Therefore, the proposed therapies combined with traditional treatments, such as chemotherapy and immunotherapy, have the potential to prolong the long‐term survival of patients with GC.

## CONFLICT OF INTEREST STATEMENT

The authors declare they have no conflicts of interest.

## Data Availability

The datasets are available from the corresponding author.
